# *catena*-Poly[[[diaqua­(1,10-phenanthroline-κ^2^ *N*,*N*′)zinc]-μ-4,4′-bipyridine-κ^2^ *N*:*N*′] dinitrate 4,4′-bipyridine hemisolvate monohydrate]

**DOI:** 10.1107/S1600536812036318

**Published:** 2012-08-31

**Authors:** Shan Xu, Yong-Cheng Dai, Qi-Ming Qiu, Qiong-Hua Jin, Cun-Lin Zhang

**Affiliations:** a Department of Chemistry, Capital Normal University, Beijing 100048, People’s Republic of China; b Key Laboratory of Terahertz Optoelectronics, Ministry of Education, Capital Normal University, Beijing 100048, People’s Republic of China

## Abstract

In the title compound, [Zn(C_10_H_8_N_2_)(C_12_H_8_N_2_)(H_2_O)_2_](NO_3_)_2_·0.5C_10_H_8_N_2_·H_2_O, the Zn^II^ atom is coordinated in a distorted octa­hedral geometry by two N atoms from two 4,4′-bipyridine (4,4′-bipy) ligands, two N atoms from a chelating 1,10-phenanthroline ligand and two O atoms from two mutually *cis* water mol­ecules. The 4,4′-bipy ligands bridge the Zn^II^ atoms into a chain structure along [100]. The uncoordinated 4,4′-bipy mol­ecule lies on an inversion center. O—H⋯O and O—H⋯N hydrogen bonds connect the cationic chains, the nitrate anions, the uncoordinated 4,4′-bipy mol­ecules and the water mol­ecules into tow-dimensional networks.

## Related literature

For background to metal complexes of 1,10-phenanthroline and its derivatives in biological systems, see: Rama Krishna *et al.* (2000 ▶); Sastri *et al.* (2003 ▶). For related structures, see: Bai *et al.* (2009 ▶); Blake *et al.* (1998 ▶); Boag *et al.* (1999 ▶); Carlucci *et al.* (1997 ▶); Chen *et al.* (2006 ▶); Du & Li (2007 ▶); Ma *et al.* (2006 ▶); Xiong *et al.* (1999 ▶); Zhang *et al.* (2009 ▶); Zhang & Janiak (2001 ▶).
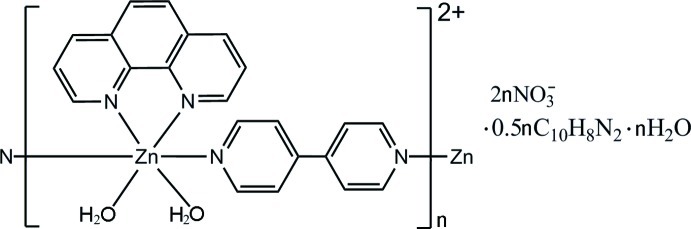


## Experimental

### 

#### Crystal data

[Zn(C_10_H_8_N_2_)(C_12_H_8_N_2_)(H_2_O)_2_](NO_3_)_2_·0.5C_10_H_8_N_2_·H_2_O
*M* *_r_* = 657.94
Monoclinic, 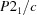

*a* = 11.3910 (11) Å
*b* = 13.0561 (13) Å
*c* = 19.8509 (18) Å
β = 103.487 (1)°
*V* = 2870.9 (5) Å^3^
*Z* = 4
Mo *K*α radiation
μ = 0.92 mm^−1^
*T* = 298 K
0.35 × 0.31 × 0.18 mm

#### Data collection

Bruker APEX CCD diffractometer
Absorption correction: multi-scan (*SADABS*; Bruker, 2001 ▶) *T*_min_ = 0.739, *T*_max_ = 0.852
14110 measured reflections
5072 independent reflections
3309 reflections with *I* > 2σ(*I*)
*R*_int_ = 0.045

#### Refinement

*R*[*F* ^2^ > 2σ(*F* ^2^)] = 0.048
*wR*(*F* ^2^) = 0.132
*S* = 1.03
5072 reflections
397 parameters
H-atom parameters constrained
Δρ_max_ = 0.81 e Å^−3^
Δρ_min_ = −0.42 e Å^−3^

### 

Data collection: *SMART* (Bruker, 2007 ▶); cell refinement: *SAINT-Plus* (Bruker, 2007 ▶); data reduction: *SAINT-Plus*; program(s) used to solve structure: *SHELXS97* (Sheldrick, 2008 ▶); program(s) used to refine structure: *SHELXL97* (Sheldrick, 2008 ▶); molecular graphics: *XP* in *SHELXTL* (Sheldrick, 2008 ▶) and *DIAMOND* (Brandenburg, 1999 ▶); software used to prepare material for publication: *SHELXTL*.

## Supplementary Material

Crystal structure: contains datablock(s) global, I. DOI: 10.1107/S1600536812036318/hy2580sup1.cif

Structure factors: contains datablock(s) I. DOI: 10.1107/S1600536812036318/hy2580Isup2.hkl

Additional supplementary materials:  crystallographic information; 3D view; checkCIF report

## Figures and Tables

**Table 1:** Hydrogen-bond geometry (Å, °)

*D*—H⋯*A*	*D*—H	H⋯*A*	*D*⋯*A*	*D*—H⋯*A*
O7—H7*C*⋯O1^i^	0.85	1.89	2.737 (5)	180
O7—H7*D*⋯O6^ii^	0.85	1.93	2.782 (5)	180
O8—H8*C*⋯N5	0.85	1.89	2.724 (5)	169
O8—H8*D*⋯O3^i^	0.85	1.90	2.744 (6)	169
O9—H9*C*⋯O2^iii^	0.85	2.24	3.091 (7)	176
O9—H9*D*⋯O4^iv^	0.85	2.27	3.114 (8)	176

Symmetry codes: (i) 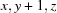; (ii) 
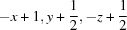
; (iii) 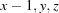; (iv) 
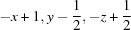
.

**Fig. 1.**
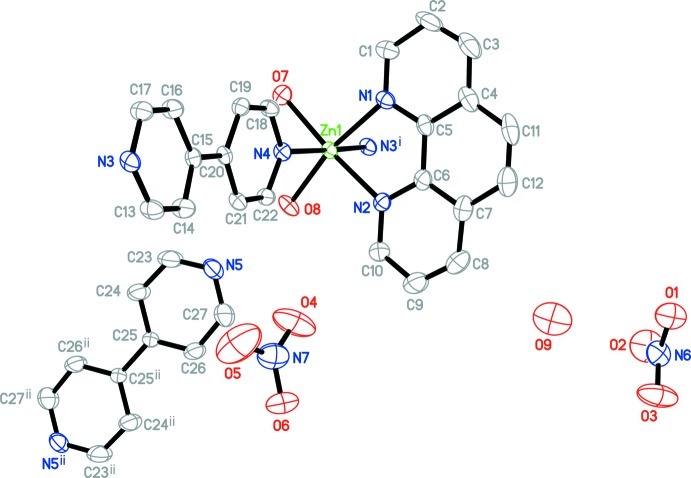
The asymmetric unit of the title compound. Displacement ellipsoids are drawn at the 30% probability level. H atoms have been omitted for clarity. [Symmetry codes: (i) 1+x, y, z; (ii) 1-x, 2-y, -z.]

**Fig. 2.**
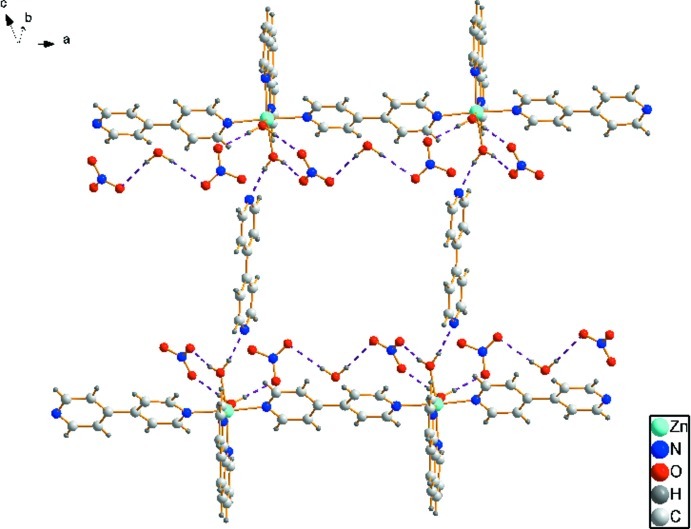
A view of the chain structure and hydrogen bonding interactions (dashed lines).

## supplementary crystallographic information

### Comment

1,10-Phenanthroline (phen) is a versatile ligand capable of forming highly stable complexes with transition metal ions (Bai *et al.*, 2009; Blake *et al.*, 1998; Chen *et al.*, 2006; Du & Li, 2007; Ma *et al.*, 2006; Zhang *et al.*, 2009; Zhang & Janiak, 2001). Metal complexes of 1,10-phenanthroline and its derivatives are interesting because they play an important role in biological systems, for example, some can recognize DNA and some can induce apoptosis in human cancer cells (Rama Krishna *et al.*, 2000; Sastri *et al.*, 2003). 4,4'-Bipyridine (4,4'-bipy) can act as a Lewis base. It can also be cocrystallized with hydrogen donors such as alcohols or transition metal complexes to form macromolecular arrays as bidentate ligands (Blake *et al.*, 1998; Boag *et al.*, 1999; Carlucci *et al.*, 1997; Du & Li, 2007; Xiong *et al.*, 1999). Here we report the structure of the title compound, a new zinc(II) complex with phen and 4,4'-bipy ligands.

In the title complex (Fig. 1), the Zn^II^ atom adopts a six-coordinated distorted octahedral geometry, where the donor atoms are two N atoms from a chelating phen ligand, two N atoms from two 4,4'-bipy ligands and two O atoms from two water molecules. The 4,4'-bipy ligands bridge the Zn^II^ atoms into a chain structure along [100] (Fig. 2). Two nitrate anions, half of a 4,4'-bipy molecule and a water molecule in the asymmetric unit are involved in the formation of O—H···O and O—H···N hydrogen bonds (Table 1). Compared with the similar comlpexes reported in literature (Bai *et al.*, 2009; Blake *et al.*, 1998; Du & Li, 2007), the Zn—N distances are longer, the Zn—O distances are shorter, and the N—Zn—N bite angles are smaller. The O—Zn—O bite angle is smaller than those in the reported zinc complexes (Bai *et al.*, 2009).

### Experimental

Zn(NO_3_)_2_ (0.2 mmol) was dissolved in 5 ml water and a hot methanolic solution (5 ml) of 4,4'-bipyridine (0.2 mmol) was added to the solution. After the mixture was stirred for 10 min, 1,10-phenanthroline (0.4 mmol) was added. The resulting solution was refluxed for 30 min and then allowed to cool to ambient temperature. The filtrate was evaporated slowly at room temperature for several weeks to yield yellow crystalline products.

### Refinement

C-bound H atoms were positioned geometrically and refined as riding atoms, with C—H = 0.93 Å and with *U*_iso_(H) = 1.2*U*_eq_(C). H atoms of water molecules were located from a difference Fourier map and refined as riding, with O—H = 0.85 Å and with *U*_iso_(H) = 1.2*U*_eq_(O).

### Crystal data

**Table:**

[Zn(C_10_H_8_N_2_)(C_12_H_8_N_2_)(H_2_O)_2_](NO_3_)_2_·0.5C_10_H_8_N_2_·H_2_O	*F*(000) = 1356
*M**_r_* = 657.94	*D*_x_ = 1.522 Mg m^−^^3^
Monoclinic, *P*2_1_/*c*	Mo *K*α radiation, λ = 0.71073 Å
Hall symbol: -P 2ybc	Cell parameters from 3365 reflections
*a* = 11.3910 (11) Å	θ = 2.4–22.8°
*b* = 13.0561 (13) Å	µ = 0.92 mm^−^^1^
*c* = 19.8509 (18) Å	*T* = 298 K
β = 103.487 (1)°	Block, yellow
*V* = 2870.9 (5) Å^3^	0.35 × 0.31 × 0.18 mm
*Z* = 4	

### Data collection

**Table:**

Bruker APEX CCD diffractometer	5072 independent reflections
Radiation source: fine-focus sealed tube	3309 reflections with *I* > 2σ(*I*)
Graphite monochromator	*R*_int_ = 0.045
φ and ω scans	θ_max_ = 25.0°, θ_min_ = 2.4°
Absorption correction: multi-scan (*SADABS*; Bruker, 2001)	*h* = −13→13
*T*_min_ = 0.739, *T*_max_ = 0.852	*k* = −15→10
14110 measured reflections	*l* = −22→23

### Refinement

**Table:**

Refinement on *F*^2^	Primary atom site location: structure-invariant direct methods
Least-squares matrix: full	Secondary atom site location: difference Fourier map
*R*[*F*^2^ > 2σ(*F*^2^)] = 0.048	Hydrogen site location: inferred from neighbouring sites
*wR*(*F*^2^) = 0.132	H-atom parameters constrained
*S* = 1.03	*w* = 1/[σ^2^(*F*_o_^2^) + (0.0523*P*)^2^ + 3.4384*P*] where *P* = (*F*_o_^2^ + 2*F*_c_^2^)/3
5072 reflections	(Δ/σ)_max_ = 0.001
397 parameters	Δρ_max_ = 0.81 e Å^−^^3^
0 restraints	Δρ_min_ = −0.42 e Å^−^^3^

### Special details

**Table:**

Geometry. All e.s.d.'s (except the e.s.d. in the dihedral angle between two l.s. planes) are estimated using the full covariance matrix. The cell e.s.d.'s are taken into account individually in the estimation of e.s.d.'s in distances, angles and torsion angles; correlations between e.s.d.'s in cell parameters are only used when they are defined by crystal symmetry. An approximate (isotropic) treatment of cell e.s.d.'s is used for estimating e.s.d.'s involving l.s. planes.
Refinement. Refinement of *F*^2^ against ALL reflections. The weighted *R*-factor *wR* and goodness of fit *S* are based on *F*^2^, conventional *R*-factors *R* are based on *F*, with *F* set to zero for negative *F*^2^. The threshold expression of *F*^2^ > σ(*F*^2^) is used only for calculating *R*-factors(gt) *etc*. and is not relevant to the choice of reflections for refinement. *R*-factors based on *F*^2^ are statistically about twice as large as those based on *F*, and *R*- factors based on ALL data will be even larger.

### Fractional atomic coordinates and isotropic or equivalent isotropic displacement parameters (Å^2^)

**Table:**

	*x*	*y*	*z*	*U*_iso_*/*U*_eq_	
Zn1	0.79800 (4)	0.79895 (4)	0.38707 (2)	0.03238 (17)	
N1	0.8386 (3)	0.7200 (3)	0.48637 (17)	0.0397 (9)	
N2	0.7735 (3)	0.6396 (3)	0.35719 (18)	0.0373 (8)	
N3	−0.0104 (3)	0.7983 (3)	0.38878 (17)	0.0363 (8)	
N4	0.6095 (3)	0.8140 (3)	0.38381 (17)	0.0337 (8)	
N5	0.6004 (4)	0.9079 (4)	0.1717 (2)	0.0576 (11)	
N6	1.0006 (4)	0.0462 (4)	0.3071 (3)	0.0659 (13)	
N7	0.3949 (6)	0.6227 (5)	0.1489 (3)	0.0878 (16)	
O1	0.9895 (4)	0.0465 (3)	0.3673 (2)	0.0837 (13)	
O2	1.0852 (4)	0.0946 (4)	0.2944 (3)	0.1126 (17)	
O3	0.9290 (5)	0.0019 (4)	0.2633 (2)	0.120 (2)	
O4	0.4873 (5)	0.6379 (5)	0.1878 (3)	0.147 (3)	
O5	0.2995 (7)	0.6575 (6)	0.1641 (4)	0.183 (3)	
O6	0.3835 (4)	0.5724 (3)	0.0954 (2)	0.0823 (13)	
O7	0.8234 (2)	0.9520 (2)	0.42605 (15)	0.0448 (8)	
H7C	0.8747	0.9816	0.4078	0.054*	
H7D	0.7603	0.9890	0.4194	0.054*	
O8	0.7743 (2)	0.8506 (2)	0.28439 (14)	0.0476 (8)	
H8C	0.7144	0.8618	0.2507	0.057*	
H8D	0.8287	0.8944	0.2826	0.057*	
O9	0.3302 (5)	0.0820 (4)	0.4013 (2)	0.1229 (18)	
H9C	0.2611	0.0847	0.3736	0.148*	
H9D	0.3830	0.0968	0.3789	0.148*	
C1	0.8733 (4)	0.7593 (4)	0.5490 (2)	0.0553 (13)	
H1	0.8777	0.8301	0.5541	0.066*	
C2	0.9036 (5)	0.6974 (6)	0.6082 (3)	0.0771 (18)	
H2	0.9285	0.7271	0.6518	0.093*	
C3	0.8967 (5)	0.5944 (6)	0.6017 (3)	0.0782 (18)	
H3	0.9166	0.5530	0.6409	0.094*	
C4	0.8602 (4)	0.5505 (5)	0.5369 (3)	0.0611 (14)	
C5	0.8325 (4)	0.6162 (4)	0.4794 (2)	0.0423 (11)	
C6	0.7960 (4)	0.5739 (4)	0.4114 (2)	0.0424 (11)	
C7	0.7839 (4)	0.4668 (4)	0.4024 (3)	0.0593 (14)	
C8	0.7443 (6)	0.4308 (5)	0.3349 (3)	0.0785 (18)	
H8	0.7346	0.3608	0.3266	0.094*	
C9	0.7204 (6)	0.4973 (5)	0.2817 (3)	0.0773 (17)	
H9	0.6925	0.4737	0.2366	0.093*	
C10	0.7374 (4)	0.6014 (4)	0.2944 (3)	0.0510 (12)	
H10	0.7226	0.6461	0.2569	0.061*	
C11	0.8484 (5)	0.4422 (5)	0.5253 (4)	0.0796 (18)	
H11	0.8674	0.3981	0.5630	0.096*	
C12	0.8109 (6)	0.4029 (5)	0.4618 (4)	0.0801 (18)	
H12	0.8023	0.3323	0.4564	0.096*	
C13	0.0304 (4)	0.7574 (4)	0.3374 (2)	0.0434 (11)	
H13	−0.0247	0.7248	0.3019	0.052*	
C14	0.1494 (4)	0.7604 (4)	0.3336 (2)	0.0442 (11)	
H14	0.1729	0.7300	0.2965	0.053*	
C15	0.2337 (3)	0.8084 (3)	0.3850 (2)	0.0315 (9)	
C16	0.1911 (3)	0.8501 (4)	0.4388 (2)	0.0426 (11)	
H16	0.2441	0.8832	0.4750	0.051*	
C17	0.0713 (4)	0.8429 (4)	0.4390 (2)	0.0428 (11)	
H17	0.0457	0.8708	0.4762	0.051*	
C18	0.5701 (3)	0.8456 (3)	0.4383 (2)	0.0366 (10)	
H18	0.6270	0.8680	0.4770	0.044*	
C19	0.4508 (3)	0.8473 (3)	0.4409 (2)	0.0350 (10)	
H19	0.4284	0.8705	0.4804	0.042*	
C20	0.3639 (3)	0.8139 (3)	0.3839 (2)	0.0308 (9)	
C21	0.4049 (3)	0.7837 (3)	0.3263 (2)	0.0360 (10)	
H21	0.3501	0.7623	0.2864	0.043*	
C22	0.5252 (3)	0.7853 (3)	0.3281 (2)	0.0358 (10)	
H22	0.5499	0.7655	0.2886	0.043*	
C23	0.5748 (6)	1.0034 (5)	0.1576 (3)	0.087 (2)	
H23	0.5841	1.0487	0.1946	0.105*	
C24	0.5350 (6)	1.0426 (4)	0.0921 (3)	0.082 (2)	
H24	0.5182	1.1122	0.0860	0.098*	
C25	0.5200 (4)	0.9801 (3)	0.0361 (2)	0.0395 (10)	
C26	0.5451 (6)	0.8796 (4)	0.0508 (3)	0.0810 (19)	
H26	0.5353	0.8320	0.0151	0.097*	
C27	0.5849 (6)	0.8484 (5)	0.1182 (3)	0.086 (2)	
H27	0.6020	0.7792	0.1261	0.103*	

### Atomic displacement parameters (Å^2^)

**Table:**

	*U*^11^	*U*^22^	*U*^33^	*U*^12^	*U*^13^	*U*^23^
Zn1	0.0222 (2)	0.0411 (3)	0.0338 (3)	0.0005 (2)	0.00634 (18)	0.0014 (2)
N1	0.0302 (18)	0.053 (3)	0.035 (2)	0.0011 (17)	0.0064 (15)	0.0012 (18)
N2	0.0286 (18)	0.039 (2)	0.044 (2)	0.0014 (16)	0.0082 (15)	−0.0020 (19)
N3	0.0218 (16)	0.050 (2)	0.0361 (19)	0.0012 (16)	0.0058 (15)	0.0011 (18)
N4	0.0244 (17)	0.042 (2)	0.0363 (19)	0.0003 (15)	0.0103 (15)	0.0024 (16)
N5	0.059 (3)	0.061 (3)	0.046 (3)	0.001 (2)	−0.001 (2)	0.009 (2)
N6	0.067 (3)	0.067 (3)	0.068 (3)	−0.014 (3)	0.024 (3)	0.014 (3)
N7	0.095 (5)	0.088 (4)	0.081 (4)	−0.027 (4)	0.022 (4)	−0.007 (4)
O1	0.085 (3)	0.093 (3)	0.078 (3)	−0.041 (2)	0.027 (2)	0.001 (2)
O2	0.106 (4)	0.127 (4)	0.118 (4)	−0.047 (3)	0.052 (3)	0.014 (3)
O3	0.137 (4)	0.156 (5)	0.063 (3)	−0.090 (4)	0.015 (3)	−0.010 (3)
O4	0.119 (4)	0.205 (7)	0.092 (4)	−0.058 (4)	−0.026 (3)	−0.014 (4)
O5	0.163 (7)	0.150 (6)	0.252 (9)	−0.036 (5)	0.081 (6)	−0.076 (6)
O6	0.100 (3)	0.084 (3)	0.068 (3)	−0.027 (2)	0.031 (2)	−0.027 (2)
O7	0.0361 (16)	0.0462 (19)	0.0530 (19)	−0.0017 (14)	0.0124 (14)	−0.0019 (16)
O8	0.0310 (16)	0.071 (2)	0.0373 (17)	−0.0045 (15)	0.0010 (13)	0.0162 (16)
O9	0.142 (5)	0.132 (5)	0.089 (4)	0.002 (4)	0.014 (3)	−0.008 (3)
C1	0.053 (3)	0.070 (4)	0.041 (3)	−0.004 (3)	0.007 (2)	−0.005 (3)
C2	0.084 (4)	0.112 (6)	0.030 (3)	0.008 (4)	0.002 (3)	0.005 (3)
C3	0.088 (5)	0.093 (5)	0.051 (4)	0.012 (4)	0.010 (3)	0.030 (4)
C4	0.056 (3)	0.073 (4)	0.054 (3)	0.011 (3)	0.012 (3)	0.023 (3)
C5	0.032 (2)	0.048 (3)	0.047 (3)	0.006 (2)	0.010 (2)	0.011 (2)
C6	0.029 (2)	0.046 (3)	0.052 (3)	0.005 (2)	0.009 (2)	0.000 (2)
C7	0.056 (3)	0.044 (3)	0.079 (4)	0.003 (2)	0.018 (3)	0.008 (3)
C8	0.103 (5)	0.042 (4)	0.086 (5)	−0.008 (3)	0.014 (4)	−0.015 (3)
C9	0.106 (5)	0.054 (4)	0.067 (4)	−0.010 (3)	0.010 (3)	−0.013 (3)
C10	0.053 (3)	0.049 (3)	0.049 (3)	−0.002 (2)	0.007 (2)	−0.004 (3)
C11	0.085 (4)	0.067 (4)	0.087 (5)	0.022 (3)	0.019 (4)	0.044 (4)
C12	0.099 (5)	0.049 (4)	0.091 (5)	0.008 (3)	0.020 (4)	0.023 (4)
C13	0.028 (2)	0.061 (3)	0.039 (2)	−0.005 (2)	0.0034 (19)	−0.009 (2)
C14	0.029 (2)	0.065 (3)	0.041 (3)	−0.002 (2)	0.0120 (19)	−0.010 (2)
C15	0.0221 (19)	0.035 (2)	0.037 (2)	0.0014 (18)	0.0067 (17)	0.004 (2)
C16	0.024 (2)	0.059 (3)	0.043 (3)	0.000 (2)	0.0042 (19)	−0.012 (2)
C17	0.030 (2)	0.063 (3)	0.037 (2)	0.003 (2)	0.0095 (19)	−0.009 (2)
C18	0.027 (2)	0.047 (3)	0.034 (2)	−0.0030 (19)	0.0032 (18)	−0.002 (2)
C19	0.030 (2)	0.041 (3)	0.036 (2)	0.0031 (19)	0.0113 (18)	−0.002 (2)
C20	0.024 (2)	0.034 (2)	0.033 (2)	0.0018 (17)	0.0050 (17)	0.0063 (19)
C21	0.030 (2)	0.045 (3)	0.031 (2)	0.0014 (19)	0.0024 (17)	0.004 (2)
C22	0.028 (2)	0.046 (3)	0.034 (2)	0.0023 (19)	0.0073 (18)	−0.004 (2)
C23	0.149 (6)	0.063 (4)	0.038 (3)	0.002 (4)	−0.002 (3)	−0.007 (3)
C24	0.156 (6)	0.038 (3)	0.040 (3)	0.006 (3)	0.000 (3)	−0.002 (3)
C25	0.040 (2)	0.037 (3)	0.037 (2)	0.0005 (19)	−0.0012 (19)	−0.006 (2)
C26	0.142 (6)	0.040 (3)	0.047 (3)	0.010 (3)	−0.006 (3)	−0.009 (3)
C27	0.132 (6)	0.048 (4)	0.063 (4)	0.013 (4)	−0.007 (4)	0.015 (3)

### Geometric parameters (Å, º)

**Table:**

Zn1—O8	2.104 (3)	C6—C7	1.412 (7)
Zn1—O7	2.138 (3)	C7—C8	1.393 (7)
Zn1—N4	2.142 (3)	C7—C12	1.418 (7)
Zn1—N2	2.163 (4)	C8—C9	1.344 (8)
Zn1—N3^i^	2.175 (3)	C8—H8	0.9300
Zn1—N1	2.176 (3)	C9—C10	1.387 (7)
N1—C1	1.317 (5)	C9—H9	0.9300
N1—C5	1.362 (6)	C10—H10	0.9300
N2—C10	1.316 (5)	C11—C12	1.335 (8)
N2—C6	1.354 (5)	C11—H11	0.9300
N3—C13	1.328 (5)	C12—H12	0.9300
N3—C17	1.329 (5)	C13—C14	1.375 (5)
N4—C18	1.329 (5)	C13—H13	0.9300
N4—C22	1.338 (5)	C14—C15	1.378 (6)
N5—C27	1.295 (7)	C14—H14	0.9300
N5—C23	1.296 (7)	C15—C16	1.383 (5)
N6—O3	1.194 (6)	C15—C20	1.490 (5)
N6—O2	1.227 (5)	C16—C17	1.369 (5)
N6—O1	1.230 (5)	C16—H16	0.9300
N7—O4	1.167 (6)	C17—H17	0.9300
N7—O6	1.229 (6)	C18—C19	1.373 (5)
N7—O5	1.278 (8)	C18—H18	0.9300
O7—H7C	0.8500	C19—C20	1.389 (5)
O7—H7D	0.8500	C19—H19	0.9300
O8—H8C	0.8500	C20—C21	1.388 (5)
O8—H8D	0.8500	C21—C22	1.363 (5)
O9—H9C	0.8500	C21—H21	0.9300
O9—H9D	0.8500	C22—H22	0.9300
C1—C2	1.401 (7)	C23—C24	1.373 (7)
C1—H1	0.9300	C23—H23	0.9300
C2—C3	1.352 (8)	C24—C25	1.357 (6)
C2—H2	0.9300	C24—H24	0.9300
C3—C4	1.381 (8)	C25—C26	1.361 (7)
C3—H3	0.9300	C25—C25^ii^	1.492 (8)
C4—C5	1.403 (6)	C26—C27	1.370 (7)
C4—C11	1.434 (8)	C26—H26	0.9300
C5—C6	1.427 (6)	C27—H27	0.9300
			
O8—Zn1—O7	91.55 (12)	C6—C7—C12	118.8 (5)
O8—Zn1—N4	92.31 (11)	C9—C8—C7	119.8 (5)
O7—Zn1—N4	88.58 (12)	C9—C8—H8	120.1
O8—Zn1—N2	93.64 (13)	C7—C8—H8	120.1
O7—Zn1—N2	174.80 (12)	C8—C9—C10	119.8 (5)
N4—Zn1—N2	90.88 (12)	C8—C9—H9	120.1
O8—Zn1—N3^i^	85.28 (11)	C10—C9—H9	120.1
O7—Zn1—N3^i^	86.98 (12)	N2—C10—C9	122.9 (5)
N4—Zn1—N3^i^	174.89 (13)	N2—C10—H10	118.5
N2—Zn1—N3^i^	93.77 (13)	C9—C10—H10	118.5
O8—Zn1—N1	168.97 (13)	C12—C11—C4	121.9 (5)
O7—Zn1—N1	97.62 (13)	C12—C11—H11	119.0
N4—Zn1—N1	94.05 (12)	C4—C11—H11	119.0
N2—Zn1—N1	77.27 (14)	C11—C12—C7	121.2 (6)
N3^i^—Zn1—N1	89.06 (12)	C11—C12—H12	119.4
C1—N1—C5	118.6 (4)	C7—C12—H12	119.4
C1—N1—Zn1	128.6 (3)	N3—C13—C14	123.8 (4)
C5—N1—Zn1	112.6 (3)	N3—C13—H13	118.1
C10—N2—C6	118.2 (4)	C14—C13—H13	118.1
C10—N2—Zn1	128.1 (3)	C13—C14—C15	119.9 (4)
C6—N2—Zn1	113.7 (3)	C13—C14—H14	120.0
C13—N3—C17	116.3 (3)	C15—C14—H14	120.0
C13—N3—Zn1^iii^	121.3 (3)	C14—C15—C16	116.1 (4)
C17—N3—Zn1^iii^	122.3 (3)	C14—C15—C20	122.4 (4)
C18—N4—C22	116.5 (3)	C16—C15—C20	121.5 (4)
C18—N4—Zn1	122.0 (3)	C17—C16—C15	120.3 (4)
C22—N4—Zn1	121.4 (3)	C17—C16—H16	119.9
C27—N5—C23	114.8 (5)	C15—C16—H16	119.9
O3—N6—O2	122.4 (5)	N3—C17—C16	123.5 (4)
O3—N6—O1	120.1 (5)	N3—C17—H17	118.2
O2—N6—O1	117.5 (5)	C16—C17—H17	118.2
O4—N7—O6	124.0 (7)	N4—C18—C19	124.2 (4)
O4—N7—O5	118.0 (7)	N4—C18—H18	117.9
O6—N7—O5	118.0 (7)	C19—C18—H18	117.9
Zn1—O7—H7C	108.6	C18—C19—C20	119.1 (4)
Zn1—O7—H7D	115.9	C18—C19—H19	120.4
H7C—O7—H7D	108.4	C20—C19—H19	120.4
Zn1—O8—H8C	135.8	C21—C20—C19	116.6 (3)
Zn1—O8—H8D	109.0	C21—C20—C15	121.7 (3)
H8C—O8—H8D	108.1	C19—C20—C15	121.7 (3)
H9C—O9—H9D	108.3	C22—C21—C20	120.3 (4)
N1—C1—C2	121.9 (5)	C22—C21—H21	119.9
N1—C1—H1	119.1	C20—C21—H21	119.9
C2—C1—H1	119.1	N4—C22—C21	123.3 (4)
C3—C2—C1	119.8 (5)	N4—C22—H22	118.3
C3—C2—H2	120.1	C21—C22—H22	118.3
C1—C2—H2	120.1	N5—C23—C24	124.8 (5)
C2—C3—C4	120.0 (5)	N5—C23—H23	117.6
C2—C3—H3	120.0	C24—C23—H23	117.6
C4—C3—H3	120.0	C25—C24—C23	120.2 (5)
C3—C4—C5	117.7 (6)	C25—C24—H24	119.9
C3—C4—C11	123.7 (6)	C23—C24—H24	119.9
C5—C4—C11	118.5 (5)	C24—C25—C26	115.1 (4)
N1—C5—C4	122.0 (5)	C24—C25—C25^ii^	121.9 (5)
N1—C5—C6	118.5 (4)	C26—C25—C25^ii^	122.9 (5)
C4—C5—C6	119.5 (5)	C25—C26—C27	120.1 (5)
N2—C6—C7	122.1 (4)	C25—C26—H26	120.0
N2—C6—C5	117.8 (4)	C27—C26—H26	120.0
C7—C6—C5	120.1 (5)	N5—C27—C26	125.0 (6)
C8—C7—C6	117.2 (5)	N5—C27—H27	117.5
C8—C7—C12	124.0 (6)	C26—C27—H27	117.5
			
O8—Zn1—N1—C1	143.1 (6)	N2—C6—C7—C8	−1.6 (7)
O7—Zn1—N1—C1	−2.8 (4)	C5—C6—C7—C8	178.1 (4)
N4—Zn1—N1—C1	−91.9 (4)	N2—C6—C7—C12	179.0 (4)
N2—Zn1—N1—C1	178.1 (4)	C5—C6—C7—C12	−1.3 (7)
N3^i^—Zn1—N1—C1	84.0 (4)	C6—C7—C8—C9	0.4 (8)
O8—Zn1—N1—C5	−32.9 (8)	C12—C7—C8—C9	179.7 (6)
O7—Zn1—N1—C5	−178.8 (3)	C7—C8—C9—C10	1.2 (10)
N4—Zn1—N1—C5	92.1 (3)	C6—N2—C10—C9	0.7 (7)
N2—Zn1—N1—C5	2.1 (3)	Zn1—N2—C10—C9	−177.4 (4)
N3^i^—Zn1—N1—C5	−92.0 (3)	C8—C9—C10—N2	−1.9 (9)
O8—Zn1—N2—C10	−9.1 (4)	C3—C4—C11—C12	178.9 (6)
N4—Zn1—N2—C10	83.2 (4)	C5—C4—C11—C12	−0.7 (9)
N3^i^—Zn1—N2—C10	−94.6 (4)	C4—C11—C12—C7	1.7 (10)
N1—Zn1—N2—C10	177.2 (4)	C8—C7—C12—C11	−180.0 (6)
O8—Zn1—N2—C6	172.7 (3)	C6—C7—C12—C11	−0.7 (9)
N4—Zn1—N2—C6	−94.9 (3)	C17—N3—C13—C14	−0.9 (7)
N3^i^—Zn1—N2—C6	87.2 (3)	Zn1^iii^—N3—C13—C14	175.9 (4)
N1—Zn1—N2—C6	−1.0 (3)	N3—C13—C14—C15	−0.4 (7)
O8—Zn1—N4—C18	−142.9 (3)	C13—C14—C15—C16	1.0 (7)
O7—Zn1—N4—C18	−51.4 (3)	C13—C14—C15—C20	179.8 (4)
N2—Zn1—N4—C18	123.4 (3)	C14—C15—C16—C17	−0.3 (7)
N1—Zn1—N4—C18	46.1 (3)	C20—C15—C16—C17	−179.2 (4)
O8—Zn1—N4—C22	41.6 (3)	C13—N3—C17—C16	1.6 (7)
O7—Zn1—N4—C22	133.1 (3)	Zn1^iii^—N3—C17—C16	−175.1 (4)
N2—Zn1—N4—C22	−52.0 (3)	C15—C16—C17—N3	−1.0 (7)
N1—Zn1—N4—C22	−129.3 (3)	C22—N4—C18—C19	2.1 (6)
C5—N1—C1—C2	−0.1 (7)	Zn1—N4—C18—C19	−173.6 (3)
Zn1—N1—C1—C2	−175.8 (4)	N4—C18—C19—C20	0.3 (7)
N1—C1—C2—C3	−0.5 (8)	C18—C19—C20—C21	−2.1 (6)
C1—C2—C3—C4	0.2 (9)	C18—C19—C20—C15	176.6 (4)
C2—C3—C4—C5	0.7 (8)	C14—C15—C20—C21	9.3 (6)
C2—C3—C4—C11	−179.0 (6)	C16—C15—C20—C21	−171.9 (4)
C1—N1—C5—C4	1.0 (6)	C14—C15—C20—C19	−169.3 (4)
Zn1—N1—C5—C4	177.4 (3)	C16—C15—C20—C19	9.5 (6)
C1—N1—C5—C6	−179.4 (4)	C19—C20—C21—C22	1.6 (6)
Zn1—N1—C5—C6	−3.0 (5)	C15—C20—C21—C22	−177.1 (4)
C3—C4—C5—N1	−1.3 (7)	C18—N4—C22—C21	−2.6 (6)
C11—C4—C5—N1	178.4 (4)	Zn1—N4—C22—C21	173.1 (3)
C3—C4—C5—C6	179.1 (4)	C20—C21—C22—N4	0.8 (6)
C11—C4—C5—C6	−1.2 (7)	C27—N5—C23—C24	−0.5 (10)
C10—N2—C6—C7	1.1 (6)	N5—C23—C24—C25	−0.2 (11)
Zn1—N2—C6—C7	179.4 (3)	C23—C24—C25—C26	1.1 (9)
C10—N2—C6—C5	−178.6 (4)	C23—C24—C25—C25^ii^	−178.4 (6)
Zn1—N2—C6—C5	−0.3 (4)	C24—C25—C26—C27	−1.3 (9)
N1—C5—C6—N2	2.3 (6)	C25^ii^—C25—C26—C27	178.1 (6)
C4—C5—C6—N2	−178.1 (4)	C23—N5—C27—C26	0.2 (10)
N1—C5—C6—C7	−177.4 (4)	C25—C26—C27—N5	0.8 (11)
C4—C5—C6—C7	2.2 (6)		

Symmetry codes: (i) *x*+1, *y*, *z*; (ii) −*x*+1, −*y*+2, −*z*; (iii) *x*−1, *y*, *z*.

### Hydrogen-bond geometry (Å, º)

**Table:**

*D*—H···*A*	*D*—H	H···*A*	*D*···*A*	*D*—H···*A*
O7—H7*C*···O1^iv^	0.85	1.89	2.737 (5)	180
O7—H7*D*···O6^v^	0.85	1.93	2.782 (5)	180
O8—H8*C*···N5	0.85	1.89	2.724 (5)	169
O8—H8*D*···O3^iv^	0.85	1.90	2.744 (6)	169
O9—H9*C*···O2^iii^	0.85	2.24	3.091 (7)	176
O9—H9*D*···O4^vi^	0.85	2.27	3.114 (8)	176

Symmetry codes: (iii) *x*−1, *y*, *z*; (iv) *x*, *y*+1, *z*; (v) −*x*+1, *y*+1/2, −*z*+1/2; (vi) −*x*+1, *y*−1/2, −*z*+1/2.
